# Metagenomic analysis of pathogenicity of *puccinia xanthii* on invasive plant *xanthium italicum*

**DOI:** 10.1038/s41598-025-18036-4

**Published:** 2025-09-29

**Authors:** Saiyaremu Halifu, Xun Deng, Li Yang, Lifeng Qian, Libin Yang

**Affiliations:** 1 The Key Laboratory of Oasis Eco-Agriculture, College of Agriculture, Xinjiang Production and Construction Corps, Shihezi University, Shihezi, 832003 People’s Republic of China; 2 Institute of Forestry Protection, Heilongjiang Forestry Academy, Harbin, 150040 People’s Republic of China; 3 Forestry and Grassland Work Station of the Xinjiang Production and Construction Corps, Urumqi, 830013 Xinjiang People’s Republic of China; 4 Institute of Nature and Ecology, Heilongjiang Academy of Sciences, Harbin, People’s Republic of China

**Keywords:** *Xanthium italicum*, *Puccinia xanthii*, Pathogenicity, Metagenomics, Ecology, Microbiology, Plant sciences, Ecology

## Abstract

Biological invasion refers to the establishment and proliferation of non-indigenous species in previously unoccupied areas, where they form wild populations. *Xanthium italicum*, an invasive species in Xinjiang, China, negatively affects local ecosystems, agriculture, and animal husbandry. In this study, we investigated the pathogenicity of *Puccinia xanthii* on the leaves of *X. italicum* by exploring the morphological characteristics of the pathogenic fungi, leaf enzyme activity measurement, photosynthesis measurement, and metagenomic sequencing. This study showed that *P. xanthii* infects the leaves of *X. italicum*, significantly reducing the activities of lyase, oxidoreductase, and antioxidant enzymes, including phenylalanine ammonia-lyase (PAL), superoxide dismutase (SOD), polyphenol oxidase (PPO), and catalase (CAT), as well as impairing photosynthesis. Furthermore, metagenomic analysis indicated that *P. xanthii* infection reduced the homogeneity and richness of phyllosphere microorganisms and increased the abundance of *P. xanthii* in the phyllosphere. Functional analysis also revealed that *P. xanthii* infection altered the diversity of microbial functions and eventually led to the development of disease symptoms and the demise of leaves through activated oxidative phosphorylation.

**Supplementary Information:**

The online version contains supplementary material available at 10.1038/s41598-025-18036-4.

## Introduction

Biological invasion is one of the most serious threats to global biodiversity and affects the economy, ecology, and social security of a region. It has become a major obstacle to biodiversity conservation, ecological safety, and sustainable agriculture development in the twenty-first century^1–3^. Against the current intensification of climate change, it is anticipated that there will be more cases of biological invasion in the future with continuous development, further aggravating the threats of biological invasion.

Invasive plant species introduced into a new environment escape from their natural enemies and symbiotic associations in their native habitats and establish new inter-species relationships with organisms in the new environment^4,5^. Based on biological groups, interspecific plant relationships can be classified into three categories: plant-plant, plant-animal, and plant-microorganism interactions^6^. Invasive plants can reduce the diversity of local edible plants through allelopathic effects in new environments, and toxic secondary metabolites in the fruits and pollen of invasive plants alter the feeding behaviors of local animals. For example, grayanotoxins in the nectar of *Rhododendron ponticum* can lead to toxicosis and paralysis in *Andrena scotica*^7^. Field experimental studies conducted by Ma et al.^8^ showed that *X. italicum*, an invasive plant species, exerts strong allelopathic effects and can reduce the biomass, carthamin A content, seed yield, 100-seed weight and seed oil content of the local plant species of Safflower by 90.04%, 33.11%, 63.89%, 40.58%, and 25.61%, respectively.

Pathogenic microorganisms play a significant role in the invasiveness of alien plant species, where such plant species carry pathogenic microorganisms that proliferate with the duration of invasion. Some pathogenic microorganisms may inhibit the growth and proliferation of the invading plant species, whereas others may suppress the growth of local plant species through overflow effects, thereby promoting their growth and proliferation. Ultimately, this process results in reduced agricultural and forestry production and disrupts the stability of ecosystems^9^. A study conducted by Chen et al.^10^ demonstrated that local pathogens accumulated asymptomatically within *Ageratina adenophora*. Fungal endophytes, as well as pathogenic fungi isolated through artificial cultivation of leaf spots from diseased *A. adenophora*, exhibited high pathogenicity towards local plant species. This indicates that *A. adenophora* has become a reservoir of pathogenic microorganisms that adversely impact the invaded ecosystems.

*Xanthium italicum*, a member of the Asteraceae family, is an annual weed species native to North America. However, this species has now spread to multiple countries and regions in Europe, Asia, and Oceania^11,12^. Since its identification in Changping District, Beijing, China in 1991, *X. italicum* has rapidly invaded various regions across China and has now spread to multiple provinces. Currently, *X. italicum* is widely distributed in various regions of Xinjiang, China^13^. With well-developed roots and strong ecological adaptability, the species exhibits a high growth rate, high seed yield, and varied seed dispersal mechanisms, and is capable of protecting itself from damage by pests and pathogens, making it a major weed species in farmlands and woodlands. Not only has *X. italicum* caused the loss of biodiversity in invaded ecosystems, but it has also exerted negative impacts on the productivity of agriculture and animal husbandry^13^. Moreover, *X. italicum* generally grows in late spring, threatening the yields of summer crops. Shao et al.^14^ reported that volatile organic compounds (VOCs; at a concentration of 0.5 μl/mL) produced by *X. italicum* significantly reduced root growth rate in *Amaranthus mangostanus* L. (by 50%), and root growth was completely inhibited at a concentration of 2.5 μL/mL. In addition, Tang et al.^15^ reported that Fresh aerial parts of *X. sibiricum* suppressed the root growth of receiver plants *Amaranthus retroflexus* L. and *Poa annua* L. by 49.1% and 69.6%, respectively. At present, the proliferation of *X. italicum* is primarily prevented and controlled through chemical-based methods and manual removal. However, manual removal is time-consuming and labor-intensive, whereas chemical-based methods are expensive and not environment friendly. Therefore, biological prevention and control methods for *X. italicum* need to be identified. A study by Wei et al.^16^ showed that three metabolites of the pathogenic fungus *Curvularia inaequalis*, namely, curvularioxide, dehydroradicinin, and radicinin, significantly inhibited the development of hypocotyls and roots of *X. italicum*, presenting effects comparable to those of the chemical herbicide glyphosate in preventing and controlling *X. italicum*.

During growth and development, plants are continuously subjected to biotic and abiotic stressors, which alter plant physiology, metabolism, and yield. Plants under the attack of pathogenic fungi resist infections through physical barriers, enzymes, and secondary metabolites, where several mechanisms play important roles^17^. Plants diseased with pathogenic fungi rapidly and transiently produce reactive oxygen species (ROS) in cells at infection sites, exerting a direct toxic effect on the pathogen. In addition, plant immunity can be induced by the production of secondary metabolites, such as phenolic compounds and flavonoids, as well as the changes in the activities of enzymes, such as phenylalanine ammonia-lyase (PAL), polyphenol oxidase (PPO), superoxide dismutase (SOD), catalase (CAT), peroxidases (POX) and glutathione reductase (GR), to resist infection^18^. As pathogens typically invade plants through stomata, host plants suppress pathogen invasion through stomatal closure. However, stomatal closure reduced the amount of CO_2_ entering chloroplasts, leading to a decrease in the rate of photosynthesis, reduced accumulation and downward transport of photosynthetic products, and ultimately resulting in the demise of the host plant due to lack of carbohydrates and other essential nutrients^19^. Baghbani et al.^20^ studied two varieties of *Zea mays* L. (MO17 and B73), and demonstrated that the pathogenic fungus *Fusarium verticillioides* significantly affected photosynthesis in the host plants. In this regard, changes in the donor side of PSII (F_v_/F_0_), minimum fluorescence (F_0_), maximum fluorescence (F_m_), and absorption flux per one active reaction center (ABS/RC) can serve as important indicators of the pathogenicity of a microbial species. Recent studies on invasive plant species have mostly focused on species distribution, risk analysis, ecological impacts, prevention, and effective utilization of resources. However, to our knowledge, studies utilizing modern techniques, such as metagenomics, to address the issue of invasive plant species are relatively scarce.

In August 2022, during an investigation on invasive species, we identified *P. xanthii* diseased *X. italicum* in the farmland shelterbelts (planted with cotton and corn) in Baojiadian Town, Manas County, Xinjiang, China. The symptoms of *X. italicum* infection were found to be consistent with those of *Xanthium strumarium* diseased with the *P. xanthii*, as supported by Seier et al.^21^. Meanwhile, field observations revealed widespread *Puccinia* infection in *X. italicum* 44° 15′ 43"N, 86° 21′ 52"E (the area with *P. xanthii* diseased *X. italicum* increased by 50% in 2023 compared to 2022). Thus, in this study, metagenomics was used to explore the accumulation and pathogenicity mechanisms of *P. xanthii* in *X. italicum*. This study not only provides a theoretical basis for preventing *X. italicum* from becoming a site for pathogen accumulation, which could disrupt the stability of the local environment, but also lays the groundwork for the development of biocontrol strategies against *X. italicum*.

## Materials and methods

### Plant material and sample collection

**Fig. 1:**
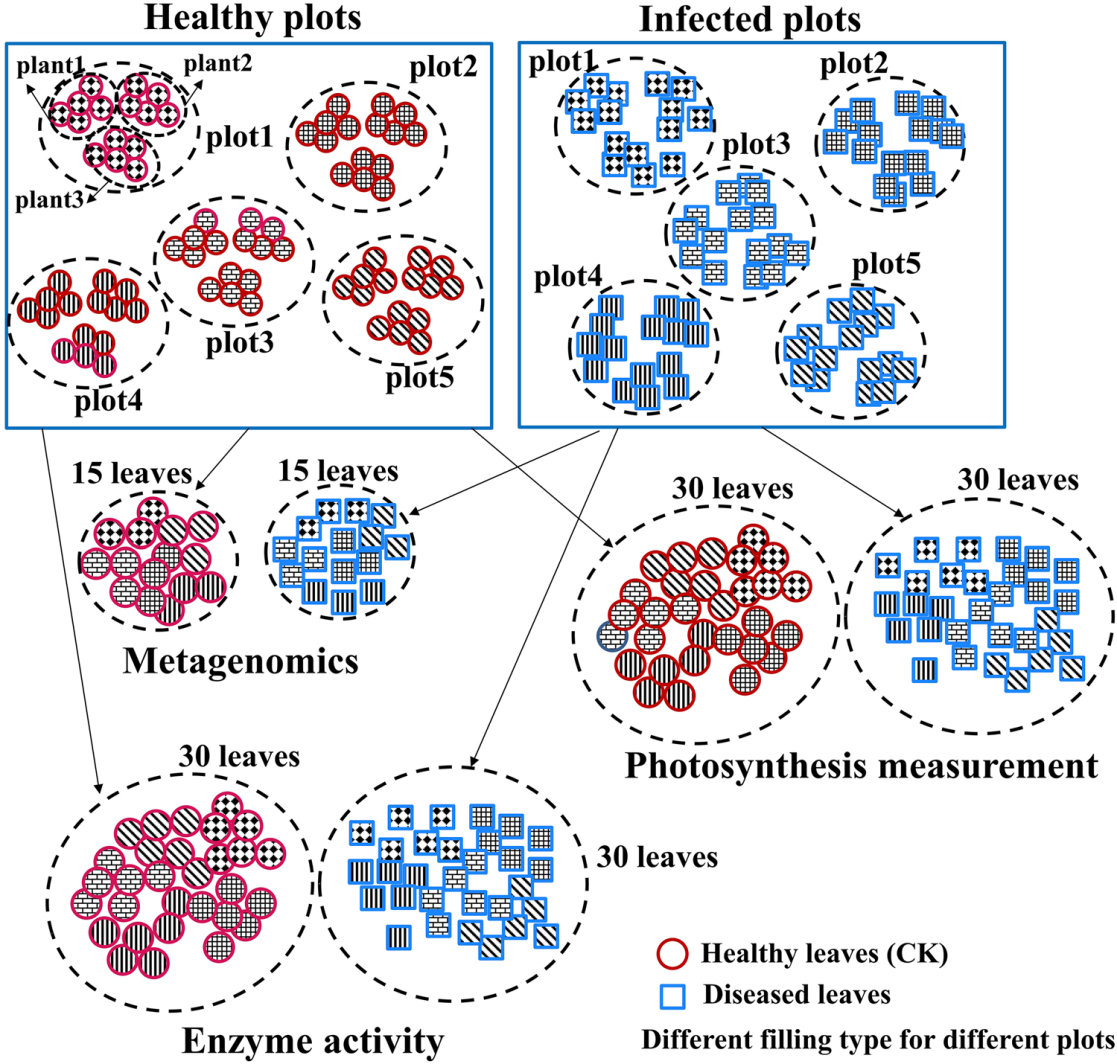
Flowchart of sample collection and analysis process. Circular shapes and square shapes represent healthy leaves and diseased leaves, respectively. Different filling types represent leaves from different sampling points.

In July 2023, 5 plots (size 5 m × 5 m) of diseased (*P. xanthii* diseased) and 5 plots of healthy *X. italicum* plants (the accession number in Chinese Virtual Herbarium is CSH0142868 identified by Jing et al.^22^ (Figure S1), and the accession number in NCBI is KX272481.1 identified by Tian et al.^23^) were individually set up based on leaf health conditions in the farmland shelterbelts on both sides of the road in Baojiadian Town, Manas County, Changji Prefecture, Xinjiang Uygur Autonomous Region (44° 15′ 43"N, 86° 21′ 52"E). The collection of *X. italicum* plants did not require specific permissions, as this species is a common weed and is not protected under national or regional legislation. The aim was to thoroughly investigate the infection situation in *X. italicum* plants and determine the diseased rate (DR) and diseased index (DI) of the leaves^24^ using the following formula:

$$DR = \frac{{\text{number of diseased leaves}}}{{\text{total number of leaves}}} \times 100$$

$$DI = \sum {[(Ni} \times i)/(N \times 4)] \times 100\%$$

where Ni represents the number of diseased leaves at each grade, i represents the grade of disease, and N represents the total number of leaves surveyed. The categorization of disease grade (i = 0, 1, 2, 3, and 4) and the corresponding disease index weight for each grade were delineated in the Supporting Information (Table S1 and S2). Next, as clearly illustrated in Fig. 1, we selected five healthy plots and five diseased plots. From each healthy plot, three healthy plants were randomly chosen, and five leaves were collected from each healthy plant, resulting in a total of 15 leaves per healthy plot. Consequently, a total of 75 healthy leaves were collected from the five healthy plots using the Five-point method. Following the same methodology, 75 diseased leaves were collected from the five diseased plots. Subsequently, we randomly selected 15 leaves from each of the healthy and disease samples, grouping them in sets of five leaves each, resulting in three sets of diseased leaves and three sets of healthy leaves. Each set is then cut into small pieces and thoroughly mixed before being stored at −80 °C for metagenomic sequencing analysis. In addition, 30 healthy leaves and 30 diseased leaves were utilized for assessing photosynthesis, while the remaining 30 healthy leaves and 30 diseased leaves were allocated for enzyme activity measurements.

### Analysis of leaf enzyme activity and photosynthetic characteristics

Healthy *X. italicum* seeds collected from the field (43° 26′ 45"N, 84° 58′ 59"E) in October 2022 were surface-sterilized with 0.5% potassium permanganate solution for 30 min., and then rinsed several times with sterile water. Mixed soils (soil: sand = 2:1) were prepared and autoclaved for sterilization. After 7 days, these soils were placed in nutrient bowls (18 cm × 18 cm) and watered thoroughly. Three days later, seeds were sown in these bowls (one seed in each bowl). Subsequently, a total of 60 nutrient bowls were placed in the experimental greenhouse at the College of Agronomy, Shihezi University, for routine care. On July 1, 2023, the seedlings were artificially diseased with the pathogen (*P. xanthii* diseased leaves collected from the field were clamped together with healthy leaves using forceps and incubated in a humid, dark chamber created with plastic films for 24 h to facilitate infection). After 40 days of the appearance of infection symptoms on leaves (60% of leaf area was diseased with the pathogen), 30 healthy and 30 diseased leaves were collected, cut into small pieces (size = 1 cm), and then mixed evenly for subsequent determination of leaf photosynthetic and enzyme activities, with three replicates for each treatment. SOD, CAT, and PPO activities were determined using colorimetry, PAL activity was determined using the hemialanine deamination method^24^. For each treatment, 30 leaves were cut into pieces (size = 1 cm) and mixed evenly through liquid nitrogen grinding, with three replicates. Photosynthetic characteristics were measured using a Li-6400XT portable photosynthesis measurement system (LI-COR, USA). We determined the photosynthetic characteristics of the uppermost, fully developed leaf of each plant at 14:00 (when the weather was suitable). With a flow rate of 500 μmol·s^−1^, control PAR of 1,300 μmol·m^−2^·s^−1^, control temperature of 25 °C, and relative humidity of 10%, 30 *Puccinia*-diseased and 30 healthy leaves were used for the analysis of photosynthetic characteristics in the sample room.

### Identification of the pathogenic fungus

Leica dissecting and DM 2000 optical microscopes (Germany) were used to observe the morphological characteristics of *P. xanthii* and measure their spore sizes, with pictures photographed. About 200 spores of *Puccinia* were picked under the dissecting microscope. Then, these spores were placed on two sterile glass slides for grinding and cell-wall breaking, after which DNA was extracted with a CTAB method. Appropriate volumes of DNA extraction solution (10 mM Tris–HCl pH 8.3, 1.5 mM MgCl_2_, 50 mM KCl, 0.01% sodium dodecyl sulfate (SDS), and 0.01% proteinase K) were used to rinse the glass slides repeatedly. The eluent was collected in a PCR tube and incubated at 37 °C for 1 h, and after a 10-min denaturation at 95 °C, it was stored under −20 °C for later PCR amplification^25^. Crude extract with a volume of 2.5 µL was used as the template of the PCR reaction system to perform the amplification (with a reaction condition of pre-denaturation at 94 °C for 3 min). Then, thirty-five cycles of amplification were conducted. A 30-s denaturation at 94 °C, followed by a 1-min annealing at 45 °C and a 1-min extension at 72 °C, was performed. Then, after a final 10-min extension at 72 °C, it was stored at 4 °C. All primers used in this study were synthesized by Shanghai Sangon Biotechnology Company (ITS5-u(5’-CAAGGTTTCTGTAGGTG-3’)/ITS4rust(5’-CAGATTACAAATTTGGGCT-3’)^26^. A phylogenetic analysis was conducted utilizing the ITS sequence data obtained from the present investigation and *Puccinia* species sourced from GenBank. A phylogenetic tree was constructed using the Maximum Likelihood (ML) method in MEGA11.

### DNA extraction and library construction

The CTAB method^26^ was used to extract DNA from leaf samples, Agarose gel electrophoresis (AGE) was performed to assess the purity and integrity of the extracted DNA, and Qubit2.0 was used for precise quantification of DNA concentration. Since DNA extraction does not involve any amplification steps, no amplification-related bias was introduced during this stage.

For metagenomic sequencing, a short-insert library (~ 350 bp) was prepared individually for each sample (i.e., one library per sample). Specifically, 1 μg of purified DNA was fragmented using Covaris ultrasonication. Library construction was carried out using the Rapid Plus DNA Library Prep Kit for Illumina (RK20208), which includes end repair, A-tailing, adapter ligation, purification, and limited-cycle PCR amplification to enrich the library fragments. Although this PCR step is necessary, we acknowledge that it may introduce amplification bias that could affect downstream metagenomic analyses, particularly in relative abundance estimations^27,28^. To mitigate the impact of such bias, all sequencing data were normalized across samples prior to statistical and compositional analysis, ensuring comparability and improving the reliability of our results.

Following PCR, each library was preliminarily quantified using Qubit 2.0 and diluted to 2 ng/μL. Insert size distribution was assessed using an Agilent 2100 Bioanalyzer (60501B; Agilent Technologies Inc., USA). The final library concentration (effective concentration > 3 nM) was precisely quantified by qPCR, which was used solely for library quantification and not for additional amplification. Then, Illumina PE150 sequencing was performed using the library.

### Quality control and annotation of sequencing data

The raw data, obtained from the Illumina Novaseq 6000 sequencing platform with a paired-end sequencing strategy, were analyzed using the Readfq (https://github.com/cjfields/readfq). Reads with proportions of low-quality bases (quality value ≤ 38) exceeding the default level of 40 bp), reads with proportions of N bases reaching the default level of 10 bp, and reads with overlaps with the adapter sequence exceeding the default value of 15 bp were excluded. In the case of host contamination among samples, reads were filtered based on alignment with the host sequences^29^. Bowtie 2 (Version 2.2.4) was used as the default software (with the following parameters: –end-to-end, –sensitive, -I 200, -X 400) to obtain clean data for subsequent analyses.

Clean reads were assembled using MEGAHIT assembly software (megahit 1.2.9, parameter: presets meta-large)^30^. Then, the assembled scaffolds were broken at their N connections, obtaining sequence fragments of Scaftigs without Ns^31^. For Scaftigs generated from single-sample assembly, fragments below 500 bp^32^ were filtered out, and further analysis and subsequent gene prediction were performed.

Based on the Scaftigs (≥ 500 bp) of all samples, MetaGeneMark^33^ was used to predict the Open Reading Frames (ORFs). Subsequently, based on the prediction results, ORFs shorter than 100 nucleotides were filtered out. CD-HIT software (Version 4.8.1)^34^ was used to remove redundancy among the prediction results of assembled ORFs in all samples, thus, obtaining an initial gene catalog without redundancy. Clustering was conducted (default parameters: identity = 95%; coverage = 90%), and the longest sequence in each cluster was selected as a representative sequence (parameters: -c 0.95, -G 0, -as 0.9, -g 1, and -d 0). Bowtie2 was then used to map the Clean Data of each sample to the initial gene catalog and calculate the number of gene reads in each sample (mapping parameters: end-to-end, sensitive, I 200, and X 400)^35^. Genes with reads ≤ 2 in all samples were filtered out to obtain the final gene catalog (Unigenes) for subsequent analysis. Based on the gene abundance information of all samples in the gene catalog, basic statistics, core-pan gene, inter-sample correlation analyses, and construction of Venn diagrams of gene numbers were conducted.

DIAMOND software (v0.9.9.110, https://github.com/bbuchfink/diamond/)^36^ was used to align Unigenes with the sequences of Bacteria, Fungi, Archaea, and Viruses extracted from the NR database (Version 2023.03) of NCBI (blastp; E-value ≤ 1e-5)^25^. After filtering, we found multiple alignment results for each sequence, resulting in multiple species classification. To ensure biological significance, the Least Common Ancestor (LCA) algorithm^37^ was used to assign the classification level before the presence of the first branch as the species annotation information of the sequence. Based on the annotation results of the LCA algorithm and gene abundance table, the abundance information of each sample at each classification level (kingdom, phylum, class, order, family, genus, and species) was obtained^38^.

DIAMOND software was used to align Unigenes with the functional database KEGG (http://www.kegg.jp/kegg/; Version 2023.02) (blastp, e value < = 1e-5)^33^. For alignment results of each sequence, the result with the highest score (one HSP > 60 bits) was selected for subsequent analysis^38^. Based on the abundance table at each classification level, gene annotation counts were summarized, relative abundance profiles were displayed, abundance clustering heat maps were generated, and NMDS dimension reduction were performed.

### Metagenomic data analysis

Origin (version 2022) software was used for single-factor analysis of variance and plotting leaf enzyme activities and photosynthetic characteristics. Based on functional abundance and comparative analysis of metabolic pathways, we performed inter-group and intra-group difference analyses using ANOSIM function from the vegan package in R (version 2.15.3). The vegan (version 2.15.3) packages of R were used to perform NMDS. Inter-group hypothesis testing using Metastats (R. version 3.1.0) was performed at each classification level (with P values). Then, the p-values were corrected using the Benjamini and Hochberg False Discovery Rate (FDR) correction, thus obtaining q-values. Furthermore, LEfSe software (v 1.0) was used to perform LEfSe analysis (with a default LDA Score of 3). Spearman’s correlation between species was estimated using the psych package of R software, and Gephi software (Gephi-0.9.5) was used to generate the network diagram with boundaries (relative threshold r > 0.6 and P < 0.05).

## Results and analyses

### Condition investigation

**Fig. 2:**
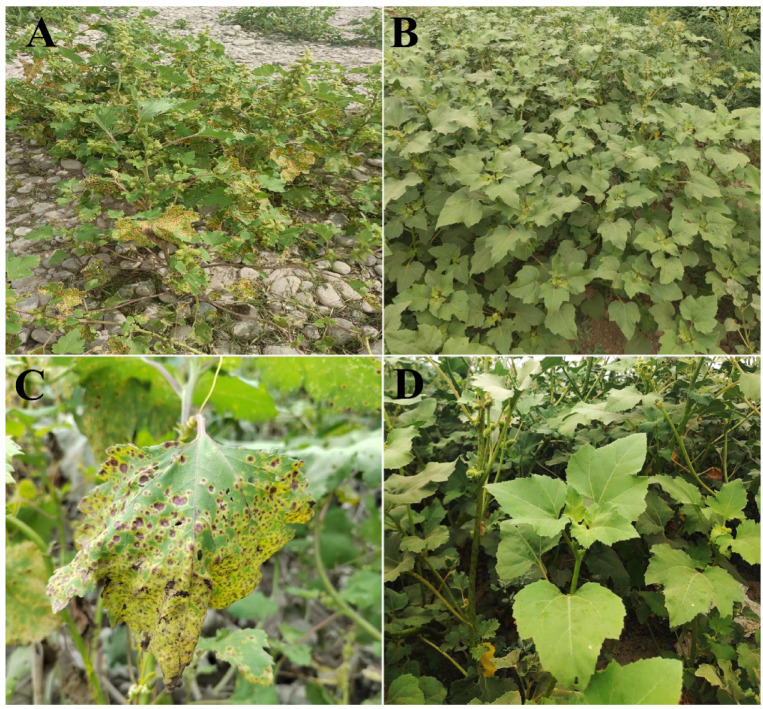
*P. xanthii* infection symptoms in *X. italicum* (collected in 2023): (**A**) Diseased plant; (**B**) Healthy plant; (**C**) Diseased leaf; (**D**) Healthy leaf.

In August 2023, *P. xanthii* diseased leaves of *X. italicum* were sampled from the shelterbelts in Manas County, Changji Prefecture, Xinjiang Uygur Autonomous Region (44.2616°N, 86.3693°E). We noted that a significant proportion of the total leaves, precisely 88.33%, were affected by *P. xanthii*. This corresponded to a DI of 80.41%, indicating a high prevalence within the studied leaves (Figs. 2A. B, C and D). Thereafter, field observations were made to investigate disease symptoms in *X. italicum*. Compared to the August 2022, the number and range of *X. italicum* leaves diseased by *P. xanthii* increased by more than 50%. Additionally, from June to July of the same year, we observed sporadic leaf infection in *X. italicum*, which escalated to more severe infections by the end of August.

### Pathogen morphology and species determination

**Fig. 3:**
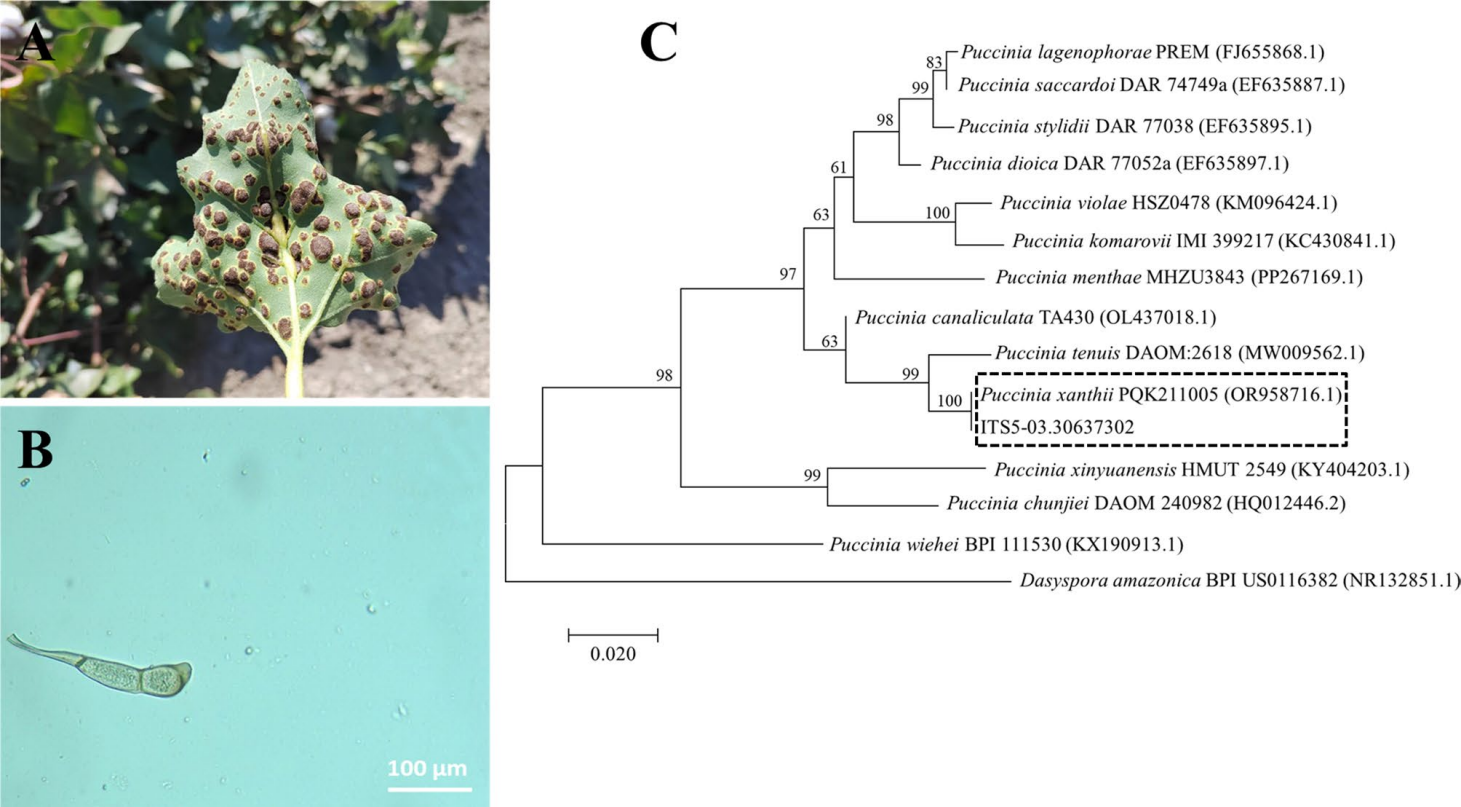
Pathogen identification: (**A**) Diseased leaf symptoms; (**B**) *P. xanthii* teliospores; (**C**) Phylogenetic relationship between *P. xanthii* isolates and some reference isolates retrieved from NCBL inferred by the ML method using the ITS regions. Bootstrap values (≥ 70%) based on 1000 replications are indicated above the branches.

After 60 days of infection in potted *X. italicum*, diseased leaves accounted for 88.33% of the total leaves, and DI of the late-stage diseased spots was 80.41%. Yellowish-brown rust-like substances were observed on the back of diseased leaves, with severely diseased leaves turning completely yellow or even defoliation (Fig. 3A). A large number of *P. xanthii* teliospore masses could be observed under an optical microscope. Each teliospore consisted of two cells with a stalk, with an elongated oval or inverted ovate shape and a size of 65.5–86.8 µm × 16.4–23 µm. The smooth teliospores were yellowish-brown or dark brown, with round tips, gradually narrowing bases, and colorless stalks with a size of 16.2–28.9 µm × 3.1–6.5 µm. A few teliospores presented slanting stalks at the bases of their lower cells (Fig. 3B). Besides, phylogenetic analysis was conducted utilizing the ITS sequence obtained from the present study and *Puccinia* species sourced from GenBank. The ML method in MEGA11 was employed for the construction of the phylogenetic tree^39^. The phylogenetic tree (Fig. 3C) revealed that the *Puccinia* isolate found on *X. italicum* grouped within a clade containing *P. xanthii*from various hosts, also corroborated by Seier et al^21^.. Therefore, both the morphological traits and genetic analysis substantiate the classification of the *Puccinia* obtained in this study as *P. xanthii*.

### Effects of P. xanthii infection on leaf enzyme activity and photosynthesis

**Fig. 4:**
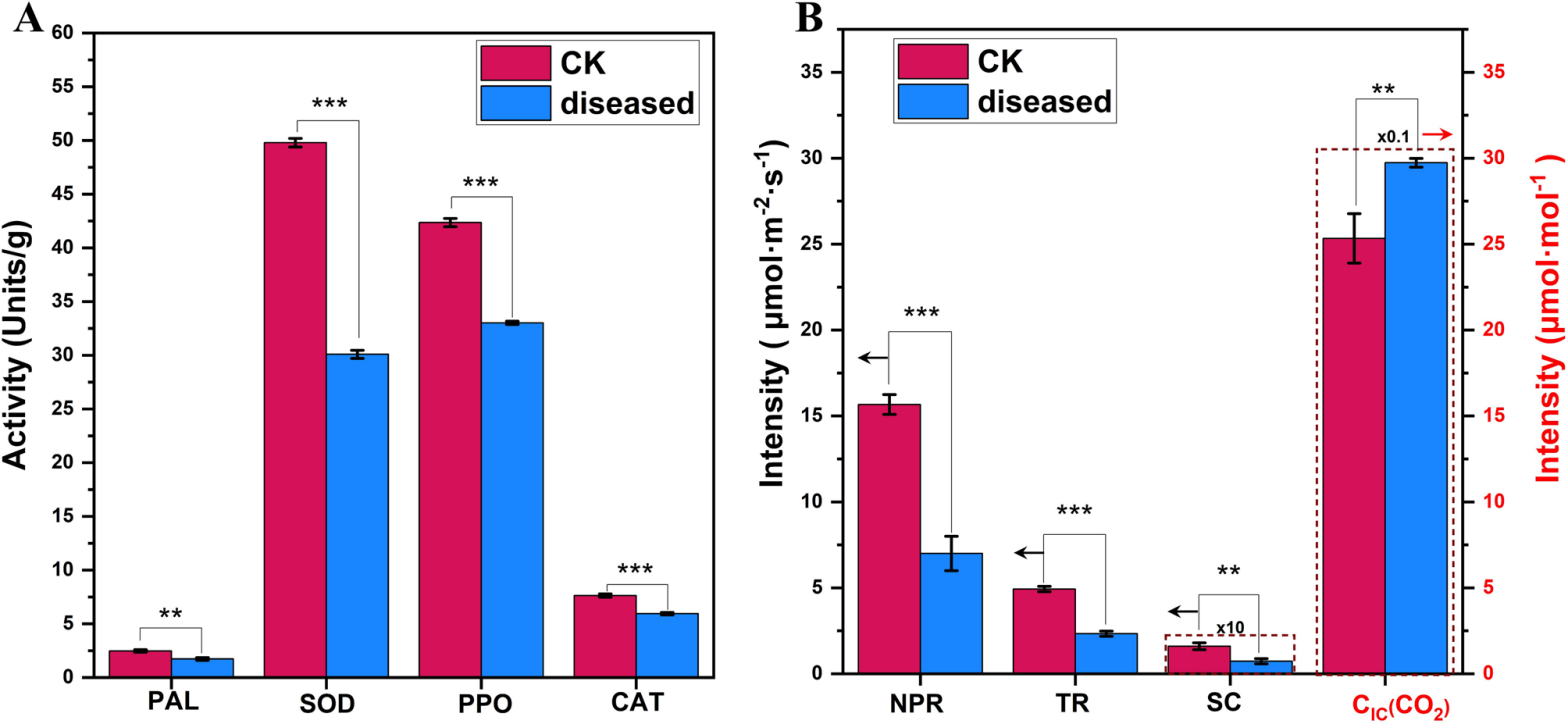
Impact of *P. xanthii* infection on leaf enzyme activity. CK: Healthy sample, diseased: Diseased sample. (**A**) and photosynthesis (**B**) in *X. italicum*. (NPR: Net photosynthetic rates, TR: transpiration rates, SC: stomatal conductance (original values × 10), C_IC_(CO_2_): intercellular carbon dioxide concentrations (original values × 0.1). The values of NPR, TR, and SC are plotted against the left y-axis (indicated by black arrows), while C_IC_(CO_2_) values are plotted against the right y-axis (indicated by red arrows).) Asterisks denote significant differences (*: p < 0.05, **: p < 0.01, ***: p < 0.001).

*P. xanthii* diseased *X. italicum* leaves exhibited significantly reduced PAL (1.74 Units/g), SOD (30.09 Units/g), PPO (33.02 Units/g), and CAT (5.95 Units/g) activities compared to healthy leaves (Fig. 4A). The activities of PAL, SOD, PPO, and CAT were reduced by 29.78%, 39.56%, 22.04%, and 22.08%, respectively. *P. xanthii* diseased *X. italicum* leaves exhibited significantly reduced photosynthesis. The net photosynthetic rate (NPR; 7.00 μmol·m^−2^·S^−1^), stomatal conductance (SC; 0.73 μmol·m^−2^·S^−1^), and transpiration rates (TR; 2.33 μmol·m^−2^·S^−1^) of diseased leaves were reduced by 55.30%, 54.40%, and 52.73%, respectively, while the intercellular CO_2_ concentration (C_ic_(CO_2_); 297.33 μmol·mol^−1^) increased by 18.77% (Fig. 4B).

### Effects of P. xanthii infection on leaf microbial community structure

**Fig. 5:**
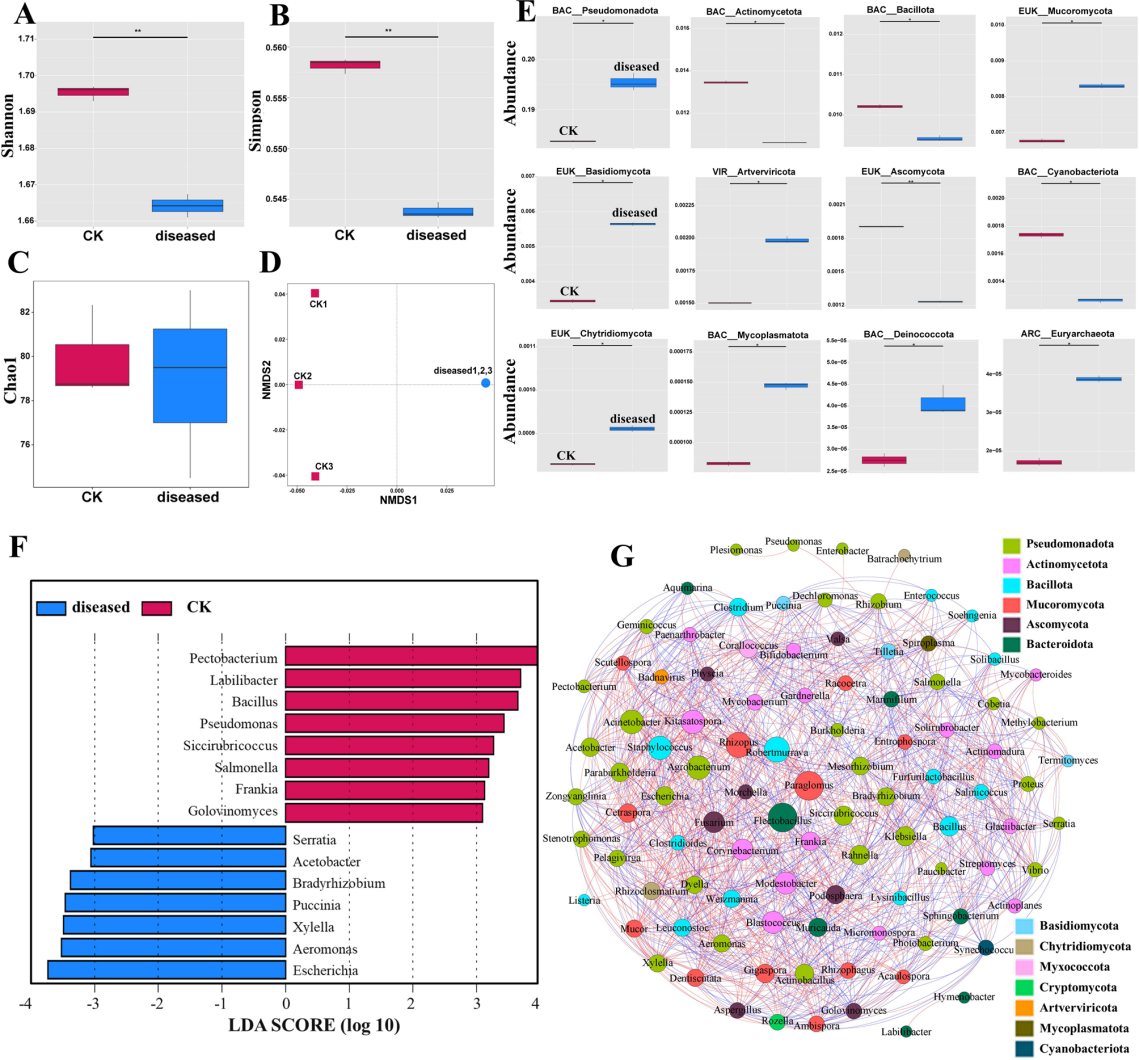
Impact of *P. xanthii* infection on phyllosphere microbial community, CK: Healthy sample, diseased: Diseased sample. (**A**) Shannon index; (**B**) Simpson index; (**C**) Chao1 index; (**D**) NMDS cluster analysis; (**E**) Differentially abundant taxa at the phylum level; (**F**) Horizontally different species; (**G**) Species ecological network analysis. Asterisks denote significant differences (*: p < 0.05, **: p < 0.01, ***: p < 0.001).

Raw Data, Clean Data and Q30 (indicating a sequencing base quality score of ≥ 30, corresponding to an error rate of ≤ 0.1%) obtained through sequencing were 5623.00–6307.19, 5617.72–6299.63, and 89.85–91.78, respectively. *P. xanthii* infection exhibited a significant impact on the phyllosphere microbial community structure of *X. italicum*. The Shannon and Simpson indexes of the diseased leaves were significantly lower than those of the healthy leaves, while the Chao1 showed no difference. This indicated that *P. xanthii* diseased samples have a reduced species abundance and evenness of phyllosphere microorganisms without any significant influence on species richness (Figs. 5A, B and C). We used Bray–Curtis distance algorithm for NMDS clustering analysis (Fig. 5D) and noted that diseased and healthy leaf samples were distributed in different quadrants with large distribution distances, indicating significant differences in the phyllosphere microbial community structures of *P. xanthii* diseased and healthy leaves.

Metastats analysis of inter-group species differences at the phylum level showed a total of 12 significantly different phyla in the samples. *P. xanthii* infection significantly increased the abundance of *Basidiomycota*, *Deinococcota*, *Mycoplasmatota*, *Pseudomonadota*, *Euryarchaeota*, *Artverviricota*, *Chytridiomycota*, and *Mucoromycota*, among which Basidiomycota members were 1.64 times highly abundant in the diseased leaves compared to healthy leaves (Fig. 5E). LEfSe analysis of inter-group species differences at the genus level (with a threshold of 3) showed 15 significantly different species in the samples. The relative abundance of *Staphylococcus*, *Corynebacterium*, *Furfurilactobacillus*, *Actinobacilus*, *Weizmannia*, *Badnavirus*, *Rhizophagus*, *Agrobacterium*, *Serratia*, *Acetobacter*, *Bradyrhizobium*, *Puccinia*, *Xylella*, *Aeromonas*, and *Escherichia* were significantly higher in diseased leaves, with increases of 20.86%, 10.55%, 28.93%, 26.27%, 17.22%, 23.80%, 18.58%, 22.87%, 22.28%, 29.12%, 8.02%, 48.93%, 57.22%, 31.09%, and 9.90%, respectively, compared to healthy leaves (Fig. 4F). After infecting the leaves of *X. italicum*, *P. xanthii* rapidly grew under favorable environments and occupied the phyllosphere microenvironments, disrupting the stable microbial community structure of the host plant. A species ecological network correlation analysis was further performed using the top 100 species at the genus level. The analysis revealed that *Paraglomus* and *Flectobacillus* were associated with 40 fungal groups, *Pseudomonas* was associated with 2 fungal groups, and *P. xanthii* was associated with 23 fungal groups (among which 12 and 11 groups exhibited positive and negative correlations with *Puccinia*, respectively.) (Fig. 4G).

### Effects of P. xanthii infection on leaf microbial functions

**Fig. 6:**
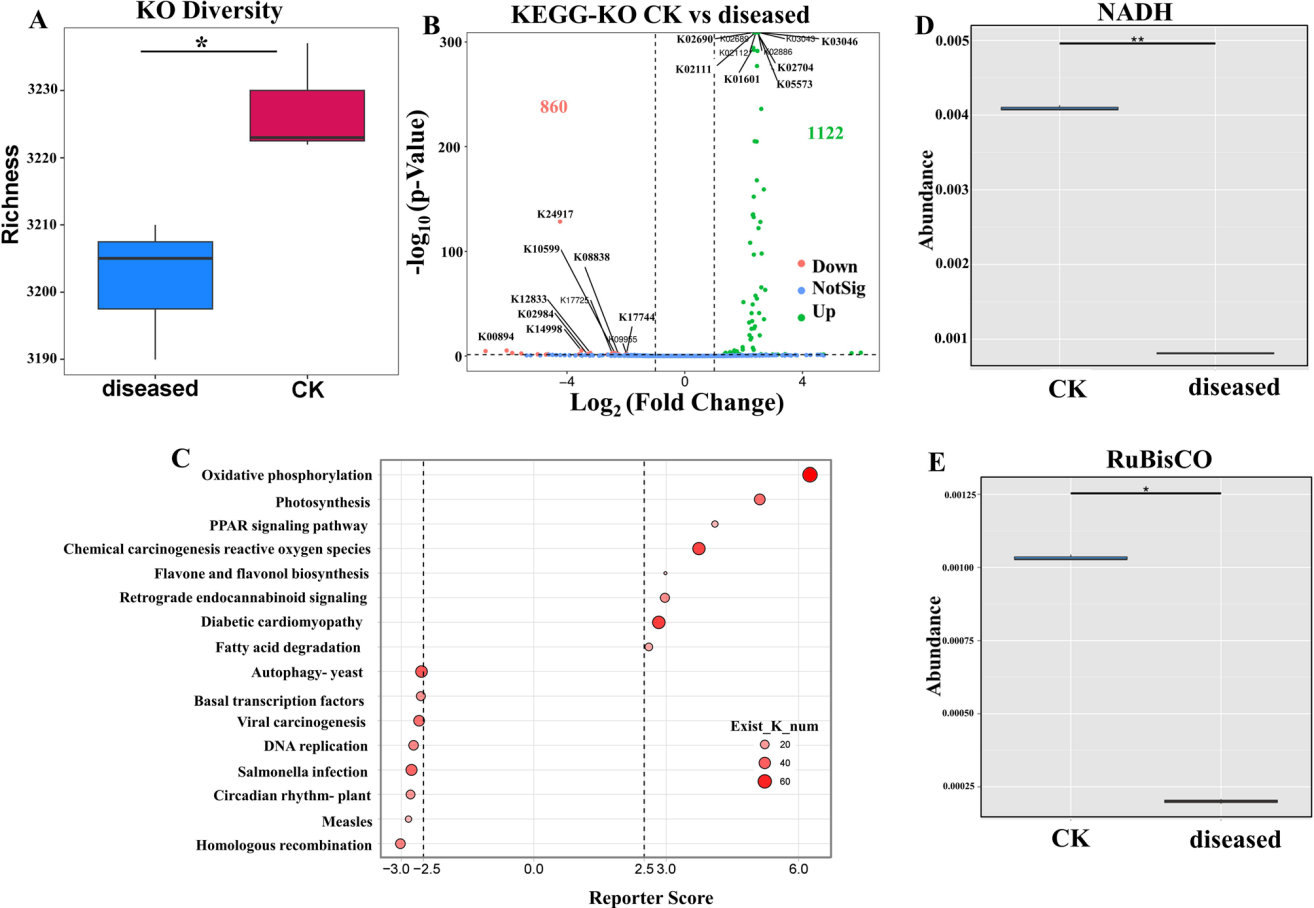
Impact of *P. xanthii* Infection on Microbial Functionalities, CK: Healthy sample, diseased: Diseased sample. (**A**) Microbial Functional Diversity Index. Richness was conducted to characterize a KO and analyzed in Kruskal–Wallis to test for differences; (**B**) Volcano Plot; (**C**). Functional Enrichment Analysis; (**D, E**) Enzyme Activities with Significant Differences. Asterisks denote significant differences (*: p < 0.05, **: p < 0.01, ***: p < 0.001).

The non-redundant encoded gene set of the phyllosphere epiphytic microorganism group was aligned with the KEGG database. Among the three groups of samples, a maximum of 3381 KOs (KEGG orthology, GhostKOALAScore > 10) were generated. *Puccinia* infection significantly reduced the functional diversity of phyllosphere microorganisms on *X. italicum* leaves (Kruskal–Wallis; p < 0.05). For the diseased leaf samples, a maximum of 3210 KOs were annotated across the three groups, which were 171 KOs less than those annotated for the healthy leaf sample (Fig. 6A). Differential enrichment analysis (DESeq2; fold change > 2 or < −2, p < 0.05, FDR < 0.1) showed that 1122 KOs were enriched in the healthy leaf sample, whereas 860 KOs were down-regulated in the diseased leaf sample (Fig. 6B). Subsequently, pathway analysis performed using the top 50 abundant KOs resulted in the annotation of 16 pathways, indicating that enriched KOs were primarily related to physiological and biochemical functions and stress resistance of plants. These included oxidative phosphorylation (223 KOs), photosynthesis (63 KOs), PPAR signaling pathway (63 KOs), chemical carcinogenesis-ROS (175 KOs), homologous recombination (72 KOs), flavone and flavonol biosynthesis (15 KOs), circadian rhythm-plant (28 KOs), salmonella infection (217 KOs), DNA replication (60 KOs), fatty acid degradation (59 KOs), basal transcription factors (36 KOs), and autophagy-yeast (82 KOs) (Fig. 6C). Metastats analysis was subsequently conducted to discern microbial functionalities, resulting in the identification of two notably distinct enzymes: NADH:ubiquinone reductase (H + -translocating) (E.C.7.1.1.2) and ribulose-bisphosphate carboxylase (RuBisCO: E.C.4.1.1.39) (Figs. 6D and 6E). E.C.7.1.1.2 is a pivotal enzyme in the electron transport chain, playing a crucial role in cellular respiration by driving ATP synthesis. E.C.4.1.1.39 serves as a fundamental enzyme in plant photosynthesis, regulating carbon dioxide fixation and converting inorganic carbon from the atmosphere into organic carbon.

### Effects of P. xanthii infection on leaf microbial functions

**Fig. 7:**
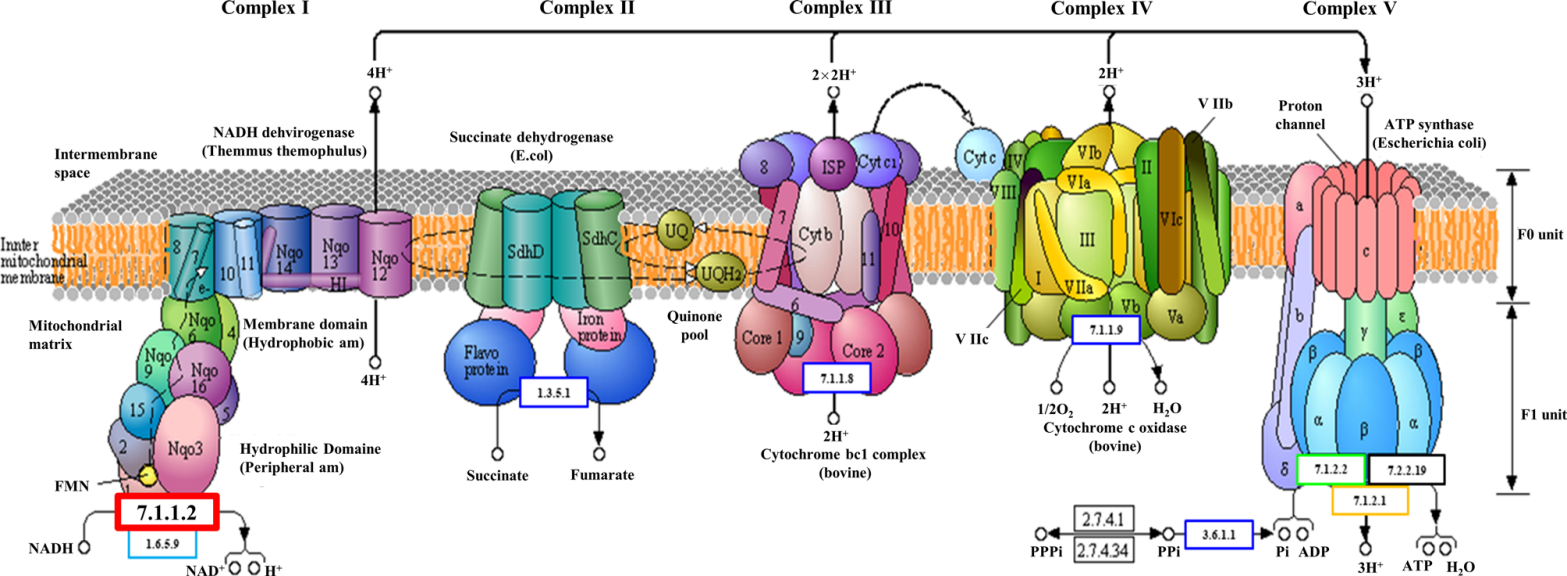
Potential impact of *P. xanthii* infection on the oxidative phosphorylation pathway of *X. italicum*. (Note: Red bold border indicates EC with significant differences; This Figure obtained according to https://www.kegg.jp/dbget-bin/www_bget?map00190)^40–42^.

Pathway annotation was performed for E.C.7.1.1.2 in order to investigate the impact of rust infection on leaf microbial functionality. E.C.7.1.1.2 was annotated to the oxidative phosphorylation pathway map00190 (Fig. 7). We also noted that the E.C.7.1.1.2 function in the diseased leaves were five-fold lower than those in the healthy leaves. The results of corresponding functional analysis through species identification tracking showed that the genus *Puccinia* was responsible for reduced E.C.7.1.1.2 functions in the map00190. This indicated that after infecting the leaves of *X. italicum*, *P. xanthii* reduced the synthesis of NADH compounds that play a critical role in the process of oxidative phosphorylation, thus reducing electron transport and ATP synthesis in leaves. Eventually, leaf cells failed to maintain energy transfer-related electron transport and ATP, which sustained cell viability, thus leading to cell death. This finding was consistent with the results of altered leaf enzyme activity and photosynthesis.

## Discussion

Wind, water, wildlife, and human activities, especially trade activities, help organisms spread to regions where they have not been historically distributed. Successful settling and proliferation of alien species in these regions can eventually threaten local ecosystems and biodiversity and cause economic losses and even risks to human health^43^. Compared to native plant species, invasive plant species are tall and exhibit larger leaf area, higher photosynthetic efficiency, and increased water and nutrient utilization, as well as rapid growth rate. In addition, these species exhibit higher root biomass ratios and extremely strong adaptive capacities to environments^44^. These advantageous traits greatly promote the spread of invasive plant species. Over the past 20 years, there has been an explosive growth in invasive species in the northwest region of China, with an average increase of 2–3 species per year, making it one of the regions with the highest number of invasive species in China and posing a threat to agricultural and forestry yields as well as ecosystem stability^4^.

*Xanthium italicum* can spread vigorously and mainly grows in farmland, riverbanks, lakes, roadsides, reservoirs, grasslands, and forest belts. This species is generally distributed in patches, forming a single dominant community and posing great threats to wetland ecosystems and agroforestry^45^. Studies by Ma et al.^46^ showed that under drought stress conditions, *X. italicum* plants in oil sunflower fields reduced seed yields (biomass), oil content, and 1,000-seed weights of sunflowers by 7.75%, 23.48%, and 13.86%, respectively, whereas under conditions of ample water supply, these percentages were elevated to 19.85%, 24.36%, and 16.19%, respectively. With the progression of alien plant invasion, local pathogenic microorganisms turn invasive plants into their habitats, thus increasing the incidence of plant diseases. Vrandecic et al.^47^ reported that sunflower (*Helianthus annuus*) stem canker is a major disease responsible for reducing sunflower yields in Croatia. During their investigation in Eastern Croatia, the group found that invasive plants such as *X. italicum*, *X. strumarium*, and *Arctium lappa* carry pathogens (such as *Diaporthe helianthi*) causing sunflower stem canker and that these invasive plant species serve as carriers and spreaders of pathogens of this disease. In sugar beet fields in Debrecen, eastern Hungary, Dávid et al.^48^ found *X. italicum* leaves, stems, and stalk diseased with the pathogen *Puccinia xanthii Schwein*. The infection rates in these plants increased with their community density, reaching up to 70% in September. Furthermore, in Dalian, China, Zhao et al.^49^ found that one-third leaves of *X. italicum* plants were diseased with *P. xanthii*. In this study, we identified *P. xanthii* diseased *X. italicum* plants in Manas County, Changji Prefecture, Xinjiang Uygur Autonomous Region. During the two-year observational period, the scale of diseased *X. italicum* plants exhibited an increasing trend. Furthermore, in late September of each year, more than 70% of *X. italicum* plants were severely diseased, resulting in leaf wilting and death. The results of pot experiments in this study verified that *P. xanthii* was responsible for the infection of *X. italicum* plants. Additionally, indoor morphological observations and molecular biology identification results verified that *P. xanthii* was the pathogen causing infection in *X. italicum* plants in the study area. Therefore, we hypothesized that *X. italicum* plants have become a habitat of *P. xanthii* in this area, exerting a significant influence on local crop production.

In August 2023, we conducted research on *P. xanthii* affected and healthy *X. italicum*, utilizing metagenomic technology to study the pathogenicity of the *P. xanthii* on *X. italicum*. The research findings indicated that the increase of *P. xanthii* infestation leads to an increase in *P. xanthii* abundance and a decrease in the diversity of *X. italicum* leaf endophytic microbial community. The phyllosphere, a unique microenvironment lacking nutrients, primarily harbors microorganisms such as fungi, bacteria, and viruses. These microorganisms either live on the leaf surfaces of host plants or inside their tissues, exhibiting an important effect on the biological processes of the host plants, including growth, development, nutrient absorption, and health status^50^. Using high-throughput sequencing technology, Chen et al.^10^ analyzed the microbial diversity in the canopy air of the invasive plant species *A. adenophora* and isolated pathogenic strains of *Didymellaceae* fungi from leaf lesions. They also found that *Didymellaceae* fungi were highly pathogenic toward 16 local plant species and pointed out that various pathogenic microorganisms in the canopy air constituted infection source that prevented the reproduction and proliferation of such invasive plant species as *A. adenophora*. Besides, Li et al.^51^ used 16S rRNA high-throughput sequencing and metagenomics to investigate citrus leaves diseased by the black-spot pathogens *Diaporthe citri* and compared them to healthy citrus leaves. Their results show that pathogen-diseased leaves present significantly reduced homogeneity of microbial communities, resulting in the emergence of a large number of new microbial communities. Simultaneously, through microbial functional annotation, researchers have found that the functions of enriched microorganisms are mostly related to siderophore competition and are associated with potential antifungal properties. Specifically, several bacterial functions related to microbiome changes will positively respond to the pathogenicity of pathogens.

Furthermore, recent studies have shown that pathogenic bacteria alter the community structure and functional diversity of leaf endophytes by occupying the phyllosphere and secreting secondary metabolites (such as toxins and hormones), and pathogenic fungi promote their settlement and effectively utilize resources in the phyllosphere^52,53^. The results presented in the above literature are consistent with our research findings. Therefore, we preliminarily believe that it may be due to the rapid increase of *P. xanthii* in the leaf endophytic environment disrupting the microbial community structure, thus potentially increasing the susceptibility of *X. italicum*. Through microbial functional diversity analysis, we found that *P. xanthii* infection significantly reduced the functional diversity of phyllospheric microorganisms in *X. italicum* plants. There were 860 KOs enriched in the diseased leaves, which were primarily correlated with physiological and biochemical functions and stress resistance of plants. A recent study showed that the pathogen *D. citri* infection reduces the functional diversity of phyllospheric microorganisms^51^. This indicates that changes in microbial functions align with alterations in the community structure of phyllospheric microorganisms, with microbial functions primarily enriched in iron complex outer membrane receptor proteins, which was similar to our results.

ROS play an important role in plant resistance against biotic and abiotic stresses. The production of ROS is crucial for the hypersensitive response of host plants to biotic and abiotic stresses that can directly disrupt the balance between the synthesis and removal of ROS. When plant cells are affected by ROS, enzymes, and chemicals, metabolites in plant cells exert a collaborative effect to protect plants from their adverse effects. Bita et al.^54^ studied pathogenic *Rhizoctonia solani* AG-3PT-stem canker of potatoes and argued that different potato varieties exhibited varying degrees of resistance to the pathogen. Among them, the variety “Savalan” exhibited the highest resistance to the disease, with POX (at 3wpi), CAT, SOD, and PAL enzyme activities increased by fourfold, 1.5-fold, 6.8-fold, and 2.7-fold, respectively, after three weeks of infection. The present study shows that *P. xanthii* infection may lead to a reduction in the activities of PAL, SOD, PPO and CAT in leaves, indicating that *P. xanthii* diseased *X. italicum* leaves could potentially exhibit decreased disease resistance.

During photosynthesis, mitochondria, the “powerhouse” of cells, help plants obtain ATP required for basic cellular functions. Notably, ATP is involved in important metabolic pathways and various enzymes are involved in the tricarboxylic acid cycle. NADH-quinone oxidoreductase, which is also referred to as NADH dehydrogenase (Complex I, NADH: quinone oxidoreductases, NQOs), is the largest and most complicated enzyme complex in mitochondria and cellular respiratory chain. NADH is oxidized to NAD^+^ through NADH-quinone oxidoreductase in the respiratory chain. The interconversions of NADH and NAD^+^ maintain redox balance in cells, ensuring stable electron transfer inside bacterial cells. The present study showed that *P. xanthii* infection significantly reduced photosynthesis of *X. italicum* plants, which was consistent with the results of microbial functional diversity analysis. Thus, *P. xanthii* infection may have an impact on oxidative phosphorylation in *X. italicum* plants, which could potentially lead to reduced efficiency in electron transfer and ATP synthesis. This may contribute to a decline in overall cell function and could be associated with the decline in health of the host plants. Besides, the subsequent analysis revealed that the enzymes EC7.1.1.2 and EC4.1.1.39 displayed significantly different levels of enzymatic activity among the various sample groups. Among them, the annotation of E.C.7.1.1.2 to the oxidative phosphorylation pathway map00190 suggests that *P. xanthii* infection could potentially disrupt the synthesis of the NADH complex. This complex is crucial for the oxidative phosphorylation process, impacting electron transport and ATP production in host leaves. Consequently, this disruption may result in leaf disease progression and ultimately lead to leaf mortality. This view is supported by a previous study on susceptible wheat varieties infected with *Puccinia triticina* and a series of near-isogenic lines carrying different Lr (leaf rust resistance) genes, including TcLr24, TcLr25, and TcLr9^55^. It needs to be noted that oxidation in Thatcher and TcLr24, TcLr25, and TcLr9 varieties depended on POXs, whereas oxidation in the TcLr26 variety depended on NADPH oxidases, indicating that different pathogen-resistant varieties may utilize different enzyme systems to initiate oxidation reactions, thus responding to the pathogenicity of pathogens. Overall, our evidence indicates that when challenged by *P. xanthii*, changes in the microbial functional diversity could lead to the appearance of disease symptoms and leaf death through the activation of oxidative phosphorylation.

## Conclusions

In this study, we have identified *P. xanthii* infection in *X. italicum* plants and used metagenomics to investigate the pathogenicity of *P. xanthii* in *X. italicum*. We found that *P. xanthii* can affect the stability of community structures of the phyllosphere microorganisms, potentially enhancing its own growth by altering spatial and nutritional dynamics. Such alterations might contribute to changes in the leaf microenvironment, which could be linked to the infection process in host plants. Moreover, after infecting the leaves, *P. xanthii* may affect oxidative phosphorylation, potentially disrupting the electron transport chain and impairing ATP synthesis, which could be associated with leaf withering and death.

## Supplementary Information

Supplementary Information.

## Data Availability

The datasets generated and/or analyzed during the current study are available in the NCBI repository, PRJNA1180918 (https://dataview.ncbi.nlm.nih.gov/object/PRJNA1180918?reviewer = ipf4112plh208jechkge6acrhb).
