# Supplementary material for: Metagenomic analysis of pathogenicity of puccinia xanthii on invasive plant xanthium italicum
Source: Sci Rep. 2025 Sep 29;15:33530. doi: 10.1038/s41598-025-18036-4 (PMC12480629; doi:10.1038/s41598-025-18036-4)
Supplement: Supplementary file 1 — Supplementary Information. [file 41598_2025_18036_MOESM1_ESM.docx]

**Supporting Information for**

**Metagenomic Analysis of Pathogenicity of *Puccinia xanthii* on Invasive Plant *Xanthium italicum***

Saiyaremu Halifu ^1,&,*^, Xun Deng ^2^, Li Yang ^3^, Lifeng Qian ^1^ and Libin Yang ^4,*^

1 The Key Laboratory of Oasis Eco-agriculture, Xinjiang Production and Construction Corps, College of Agri-culture, Shihezi University, Shihezi 832003, People's Republic of China; 437246661@qq.com (Saiyaremu Halifu), Q253416@126.com (Lifeng Qian)

2 Institute of Forestry Protection, Heilongjiang Forestry Academy, Harbin 150040, People’s Republic of China; dengxun1125@163.com

3 Forestry and Grassland Work Station of the Xinjiang Production and Construction Corps, Urumqi 830013, Xinjiang, People's Republic of China; 980999523@qq.com

4 Institute of Nature and Ecology, Heilongjiang Academy of Sciences, Harbin, People’s Republic of China; 13664600518@139.com

*Correspondence: 437246661@qq.com, 13664600518@139.com.

**Figure S1** The specimen of *Xanthium italicum* is deposited in the Chinese Virtual Herbarium with the accession number CSH0142868.

**
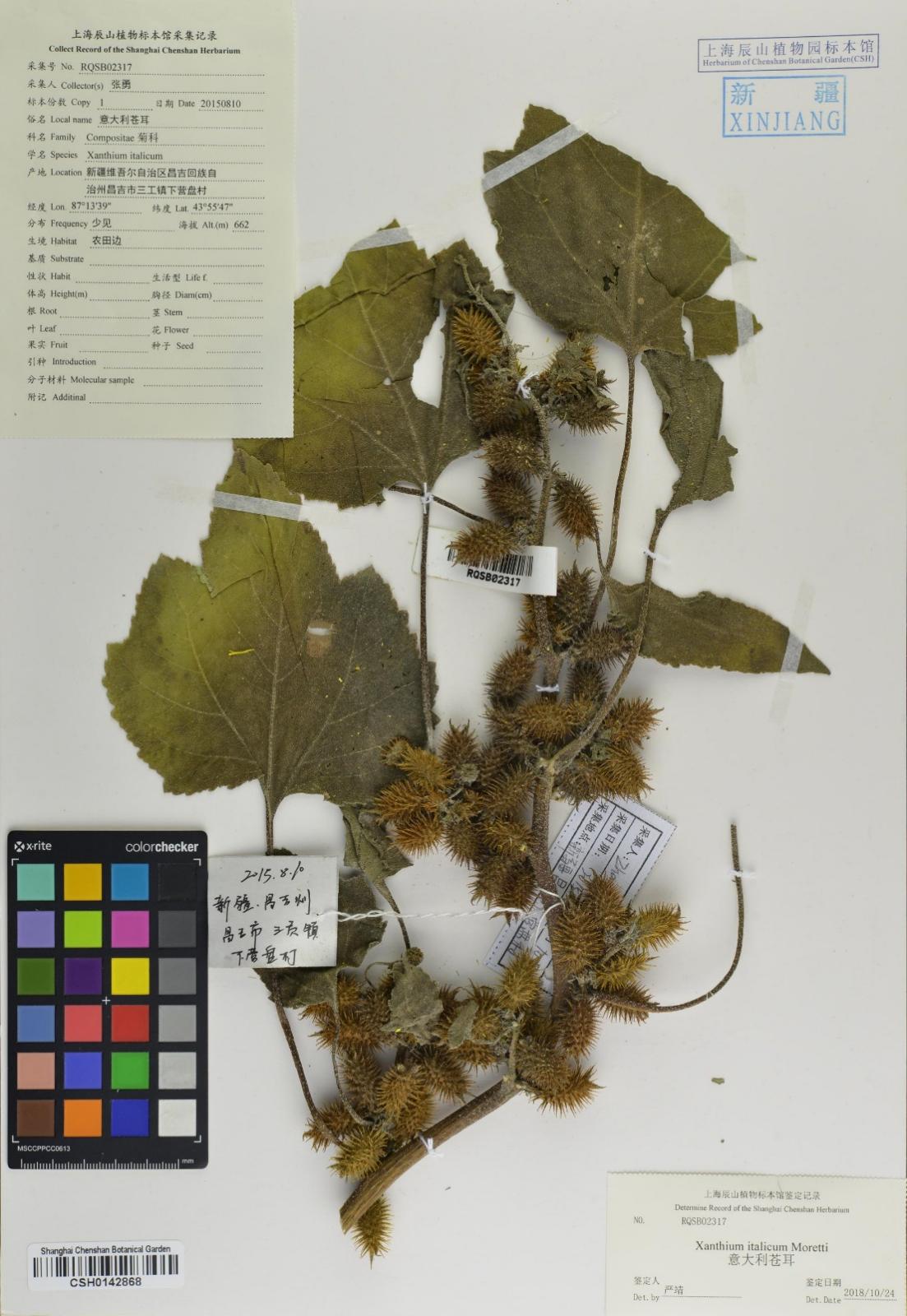
**

**Table S1** Disease resistance identification grading criteria

| Disease classification (i) | Symptoms |
| --- | --- |
| 0 | No symptoms |
| 1 | The proportion of the leaf surface area affected by disease lesions accounted for 0% to 25%. |
| 2 | The proportion of the leaf surface area affected by disease lesions accounted for 25% to 50%. |
| 3 | The proportion of the leaf surface area affected by disease lesions accounted for 50% to 75%. |
| 4 | The proportion of the leaf surface area affected by disease lesions exceeded 75%. |

**Table S2** Incidence of all levels of disease

| Disease classification (i) | Diseased rate (%) | Diseased index (%) |
| --- | --- | --- |
| 0 | 11.67 | 0 |
| 1 | 6.67 | 1.67 |
| 2 | 3.33 | 3.33 |
| 3 | 5.00 | 7.08 |
| 4 | 73.33 | 80.42 |
